# Supplementary material for: Intestinal Ketogenesis and Permeability
Source: Int J Mol Sci. 2024 Jun 14;25(12):6555. doi: 10.3390/ijms25126555 (PMC11204016; doi:10.3390/ijms25126555)
Supplement: Supplementary file 1 [file ijms-25-06555-s001.zip › ijms-2991709-supplementary.pdf]

Supplementary method

Endotoxin measurement

Cell culture. The mouse macrophage cell lines J774.2 and RAW264.7 were purchased from SigmaAldrich (Stockholm, Sweden; cat. no. 85011428 and 91062702) and the cells were cultured in Dulbecco's Modified Eagle's Medium with 10% fetal bovine serum, 1% non-essential amino acids, and 100 IU/ml penicillin-streptomycin. All cell culture products were purchased from Life Technologies Invitrogen AB, Lidingö, Sweden. Cells were grown in a humidified atmosphere at 5% CO_2 and 37 °C. The macrophages were grown to a confluence near but below 95%, after which the cells were scraped loose and seeded onto 24-well cell culture plates at a density of 0.5×10^6 cells/well.

Nitic oxide and chemiluminescence analysis. Before initiating the experiments, a standard curve was performed with known concentrations of nitric oxide (NO) by stimulation the macrophages with LPS. The cells were stimulated by 0–100 ng/mL of LPS (SigmaAldrich) and 0.2 mM L-arginine (SigmaAldrich) during 16 h. The nitric oxide concentration was then determined by adding 0.2mL supernatant with 1mL HCL in a tight-fitting tube, shaken and the equilibrated gas then immediately injected into the sample line of a chemiluminescence NO analyzer (Modified Seres NOX 4000, Seres, Aix-en-Provence, France). The detection limit for NO was 1 ppb and calibrations were performed with known concentrations of NO in N_2 (AGA, Stockholm, Sweden).

Evaluation of samples for LPS. The macrophage cells were stimulated by basolateral medium from the Caco-2 cells experiments (describe above) treated by short-chain fatty acid butyrate (10 mM), HMGCS2 inhibitor hymeglusin (1 µM), LPS (1µg/mL) and a cocktail of fats (cholesterol (0.05mM), 2-monooleoylglycerol (0.2mM), L-∝-Lysophosphatidyl choline (0.2mM) and Oleic acid (0.6mM) over 16 h.

Time-matched vehicle-treated samples served as control. Experiments were performed in triplicates, and each experiment was repeated at least twice. After the 16 h-treatment, the cell count and viability were estimated using trypan blue staining and a TC20TM Automated Cell Counter (BioRad Laboratories, Hercules CA, USA).

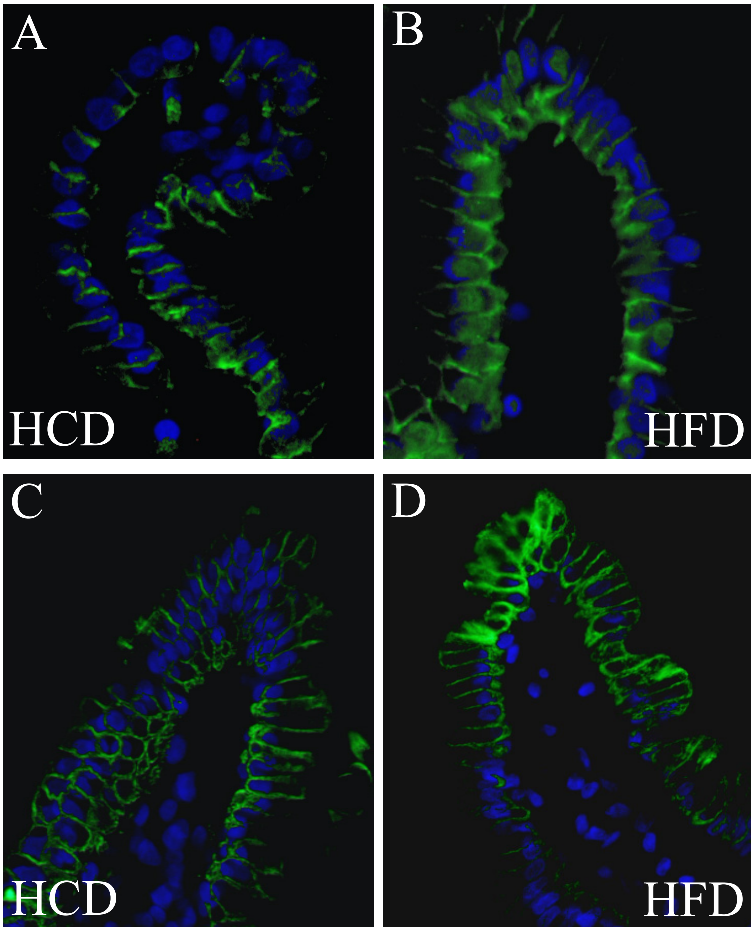

Supplementary Figure S1

Representative immunofluorescence staining of Claudin 3 (green) (A-B), and Cytokeratin 8 (green) (C-D) in human jejunal mucosa after a two-week high-carbohydrate diet (HCD) or high-fat diet (HFD). Blue staining: nucleus. Each protein is represented with staining from one subject. Original magnification x20.
